# Immunotherapy-induced cytotoxic T follicular helper cells reduce numbers of retrovirus-infected reservoir cells in B cell follicles

**DOI:** 10.1371/journal.ppat.1011725

**Published:** 2023-10-26

**Authors:** Anna Malyshkina, Wibke Bayer, Philip Podschwadt, Lucas Otto, Zehra Karakoese, Kathrin Sutter, Kirsten Bruderek, Baoxiao Wang, Kerry J. Lavender, Mario L. Santiago, Pia Madeleine Leipe, Carina Elsner, Stefan Esser, Sven Brandau, Matthias Gunzer, Ulf Dittmer

**Affiliations:** 1 Institute for Virology, University Hospital Essen, University of Duisburg-Essen, Essen, Germany; 2 Institute for Experimental Immunology and Imaging, University Hospital Essen, University of Duisburg-Essen, Essen, Germany; 3 Institute for Translational HIV Research, University of Duisburg-Essen, Essen, Germany; 4 Department of Otorhinolaryngology, Head and Neck Surgery, University Hospital Essen, University of Duisburg-Essen, Essen, Germany; 5 Department of Biochemistry, Microbiology and Immunology, College of Medicine, University of Saskatchewan, Saskatoon, Saskatchewan, Canada; 6 Division of Infectious Diseases, Department of Medicine, University of Colorado Anschutz Medical Campus, Aurora, Colorado, United States of America; Vaccine Research Center, UNITED STATES

## Abstract

Antiretroviral therapy (ART) transformed HIV from a life-threatening disease to a chronic condition. However, eliminating the virus remains an elusive therapy goal. For several decades, Friend virus (FV) infection serves as a murine model to study retrovirus immunity. Similar to HIV, FV persists at low levels in lymph nodes B cell follicles avoiding elimination by immune cells. Such immune-privileged reservoirs exclude cytotoxic T cells from entry. However, CXCR5^+^ T cells are permitted to traffic through germinal centers. This marker is predominantly expressed by CD4^+^ follicular helper T cells (Tfh). Therefore, we explored immunotherapy to induce cytotoxic Tfh, which are rarely found under physiological conditions. The TNF receptor family member CD137 was first identified as a promising target for cancer immunotherapy. We demonstrated that FV-infected mice treatment with αCD137 antibody resulted in an induction of the cytotoxic program in Tfh. The therapy significantly increased numbers of cytotoxic Tfh within B cell follicles and contributed to viral load reduction. Moreover, αCD137 antibody combined with ART delayed virus rebound upon treatment termination without disturbing the lymph node architecture or antibody responses. Thus, αCD137 antibody therapy might be a novel strategy to target the retroviral reservoir and an interesting approach for HIV cure research.

## Author summary

Despite the development of potent antiretroviral therapy, elimination of the HIV reservoir from lymph nodes remains elusive. The main reason is that lymph nodes are an immune privileged site in which cytotoxic T cell activity is restricted. We used the Friend retrovirus (FV) mouse model to develop a novel therapy approach that targets the retroviral reservoir in lymph nodes. Immunotherapy with CD137 activating antibodies induced a cytotoxic program in follicular CD4^+^ T cells, which improved their killing capacity and resulted in a reduction of the FV reservoir in lymph nodes. Antibody treatment combined with antiretroviral therapy delayed virus rebound upon treatment termination without disturbing the lymph node architecture or antibody responses to unrelated antigens. Thus, αCD137 antibody therapy might be a novel strategy to target the retroviral reservoir in lymph nodes and an interesting approach for HIV cure research.

## Introduction

Since antiretroviral therapy (ART) has been introduced to the clinic, infection with human immunodeficiency virus (HIV) transformed from a life-threatening disease to a chronic condition. However, for individuals on ART, the therapy goal to eliminate the virus completely or reach functional cure, when HIV does not reactivate after therapy termination, continues to be elusive. According to recent studies, HIV-infected CD4^+^ T cells “hide” in the B cell follicles of lymph nodes where they persist during ART [1]. Conventional CD8^+^ T lymphocytes are not eligible to enter B cell follicles and therefore unable to kill these infected targets. Recently identified follicular CD8^+^ T cells may be involved in HIV control; however, they have less cytotoxic potential than their conventional counterparts [2]. Some of these follicular CD8^+^ cells have a regulatory phenotype and a therefore less efficient killers than extrafollicular CD8^+^ T cells [3]. Thus, HIV-infected follicular helper T cells form an inaccessible reservoir in lymph nodes and spleen that can so far not be eradicated by any clinically used or experimentally tested antiretroviral therapy.

For several decades, Friend virus (FV) infection has served as a murine model to study retroviruses and their interaction with cells of the immune system [4]. FV can induce acute leukemia in susceptible mice, but resistant mouse strains, like C57BL/6 mice, control acute virus replication and develop a life-long chronic infection [5]. Similar to HIV, FV is not completely eliminated by immune cells during the acute phase of infection, but persists at low levels in spleen and lymph nodes forming a viral reservoir [6]. This reservoir consists of infected follicular CD4^+^ T cells and follicular B cells [6]. Thus, in analogy to HIV the Friend viral reservoir is located in germinal centers [7–9]. The main reason for this special location of the virus is that germinal centers are immune privileged sites that exclude cytotoxic T cells from entry [10]. Accordingly, when cytotoxic CD8^+^ T cells are experimentally depleted in FV-infected mice, reservoir virus redistributes from germinal centers and spreads systemically [6]. Interestingly, during the chronic phase of FV infection, a subpopulation of CD4^+^ T cells develops cytotoxic activity via FasL expression and keeps the chronic virus in check. However, these CD4^+^ T cells can also not enter B cell follicles nor eliminate the viral reservoir, because they do not express the key entry marker CXCR5^+^ [11].

CXCR5 is predominantly expressed by CD4^+^ follicular helper T (Tfh) cells, which, of course, are permitted to traffic through germinal centers. Although there is some evidence that Tfh with cytotoxic activity may exist in very small numbers [12], they very likely do not play a major role in restricting the reservoir of retroviruses. Therefore, exploring therapeutic interventions to augment the cytotoxicity of CXCR5^+^ Tfh is an interesting novel approach to eradicate retroviruses from their reservoir.

CD137 is a tumor necrosis factor (TNF) receptor family member that is central for the initiation of the cytotoxic exocytosis pathway in T cells [13]. Signaling via this receptor induces the transcription factor Eomesodermin (Eomes), which in turn facilitates Perforin (Perf) and Granzyme (Gzm) expression [14]. Impressive results of tumor elimination due to the improvement of cytotoxic T cell functions after the antibody administration were recently described in mouse and human studies [15–21]. In our hands, immunotherapy with an agonistic antibody against mouse CD137 induced tumor rejection of FV-induced leukemia cells in chronically FV-infected mice [22]. This was associated with augmented CD4^+^ T cell cytotoxicity that depended on the exocytosis pathway of target cell killing.

Thus, we addressed the hypothesis that αCD137 therapy might generate cytotoxic Tfh cells that target the retroviral reservoir. In the current study we show that cytotoxic Tfh were indeed induced upon therapy in FV-infected mice and that infected reservoir cells could be completely eliminated in many B cell follicles. Also, in FV-infected mice under ART, αCD137 therapy induced cytotoxic CD4^+^ Tfh cells that reduced the reservoir size. Here, viral load rebound following therapy termination was significantly reduced in antibody-receiving mice. These results demonstrate that the induction of CXCR5^+^ Tfh CD4^+^ T cell cytotoxicity is an interesting novel approach to eradicate retroviruses from their reservoir.

## Results

### αCD137 therapy induces cytotoxic Tfh cells in a retrovirus infection model

**Fig 1:**
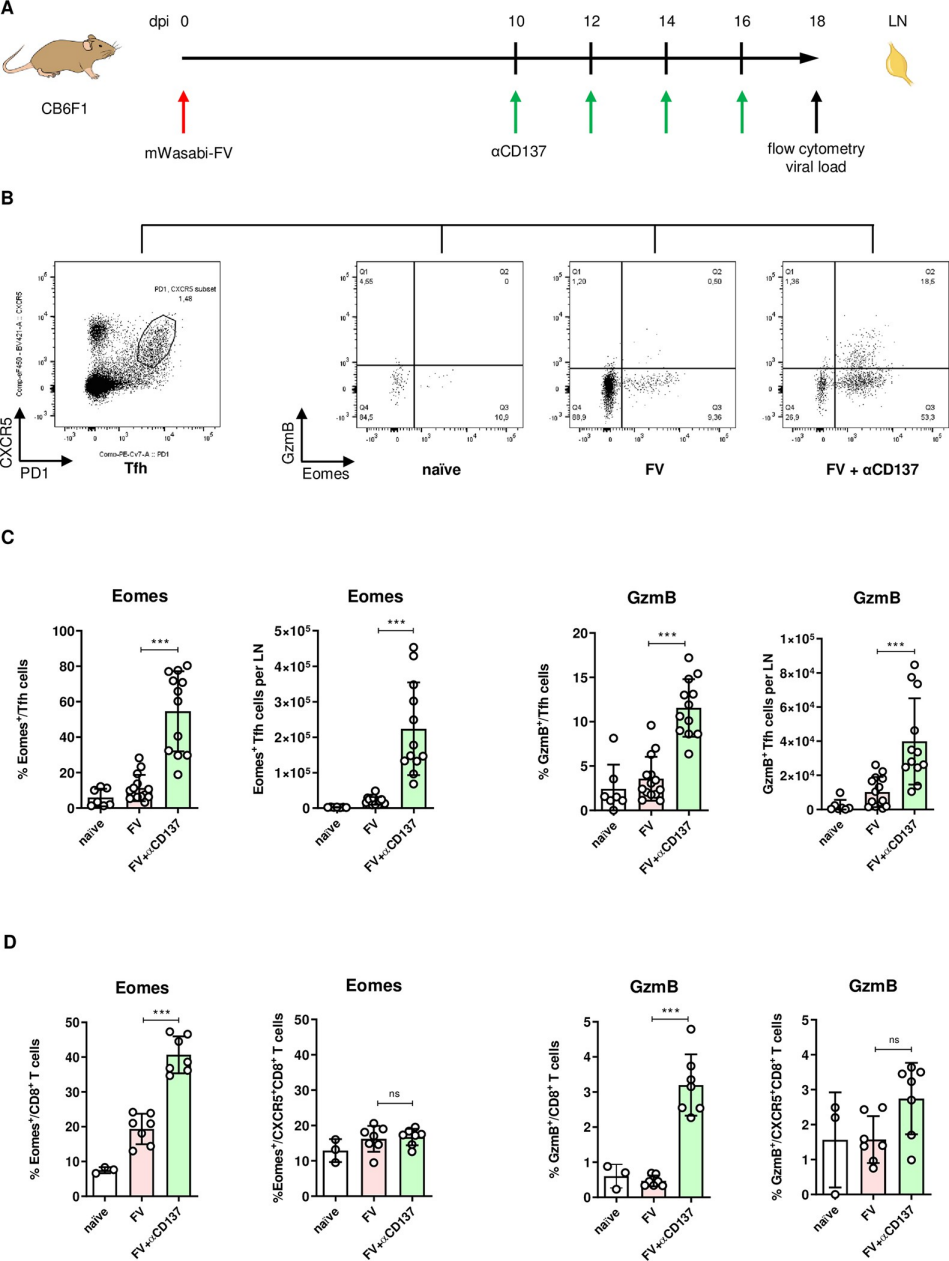
αCD137 therapy induces cytotoxic Tfh in FV-infected mice. CB6F1 mice were infected with mWasabi-expressing FV, αCD137-treated from 10–16 dpi, and sacrificed on day 18 pi (A). The lymph nodes (LN) were used to establish single-cell suspensions. (B) CXCR5 and PD1 co-expression was used to characterize Tfh cells. Representative dot plots indicate the expression of cytotoxic molecules (Eomes and GzmB) by CXCR5^+^ PD1^+^ CD4^+^ T cells. Frequencies and absolute numbers per lymph node (LN) of Eomes^+^ or GzmB^+^ Tfh cells (C) or CD8^+^ T cells (D) are shown. Dots indicate values from individual mice. Median of the groups ± SD is given; ns–not significant, ***p < 0.001, one-way ANOVA.

First, we analyzed whether Tfh cells start to express cytotoxic molecules in FV-infected mice after αCD137 treatment. For this purpose, we infected CB6F1 mice with recombinant FV, expressing the fluorescent protein mWasabi in infected cells and subsequently administered 100 μg of the αCD137 antibody four times starting on day 10 post infection (pi). The experimental schedule is shown in Fig 1A. We showed previously that the reservoir of FV is established within a few days of infection [6], therefore waiting 10 days was sufficient to study treatment effects on the viral reservoir. Additionally, we required CD4^+^ T cells to be primed by the time of therapy initiation. It was shown by our group and others that FV-reactive CD4^+^ T cells can be detected as early as 4 days after FV infection [23], peaking around day 9 pi [24].

Thus, starting the therapy on day 10 pi allowed us to immunomodulate responding effector CD4^+^ T cells.

On day 18 pi, we performed flow cytometric characterization of Tfh cells from lymph nodes. Tfh cells were defined as CXCR5^+^ PD1^+^ FoxP3^-^ CD4^+^ T cells. PD1, a well-known marker of T cell exhaustion, in conjunction with CXCR5 is commonly used to identify and characterize Tfh cells [25]. Follicular T regulatory cells (Foxp3^+^) were excluded from the analysis. To study cytotoxicity markers, we determined the Eomes and GzmB expression by Tfh cells. It was not surprising that only very low frequencies of Tfh cells expressing Eomes and GzmB were found in infected but untreated mice, since we previously showed that cytotoxic CD4^+^ T cells are rare during acute FV infection [26]. However, in the group of mice receiving the therapy, both the frequencies and absolute numbers of Eomes- and GzmB-expressing Tfh cells significantly increased (Fig 1B and 1C). Thus, CD4^+^ Tfh cells developed a cytotoxic phenotype after αCD137 therapy and may be able to reduce or eliminate FV-infected cells from germinal centers.

Similar induction of cytotoxicity was found in CD4^+^ T cells after peripheral blood mononuclear cells (PBMC) from healthy human volunteers were stimulated with anti-human CD137 agonist (BPS Biosciences, San Diego, USA). For these experiments, we quantified CD107a expression as a well-established marker of cytotoxic degranulation [27, 28]. Due to the very low frequency of peripheral Tfh cells within PBMC [29], we analyzed total CD4^+^ T cells. We detected significantly higher frequencies of CD107a expressing CD4^+^ T cells after antibody stimulation compared to untreated cells in samples from five donors (S1 Fig).

CD137 co-stimulation can similarly augment CD8^+^ T cell cytotoxicity as shown for infectious diseases and cancer [30–32]. Accordingly, we observed that conventional CD8^+^ T cells expressing Eomes and GzmB were increased in frequencies after αCD137 therapy, but this was not the case for the subpopulation of CXCR5^+^ follicular CD8^+^ T cells, which remained at very low percentages after antibody administration (Fig 1D). Therefore, follicular CD8^+^ T cells were very likely not sufficiently high in numbers to affect the viral reservoir.

### Cytotoxic Tfh cells, induced by αCD137 therapy, are present in B cell follicles

**Fig 2:**
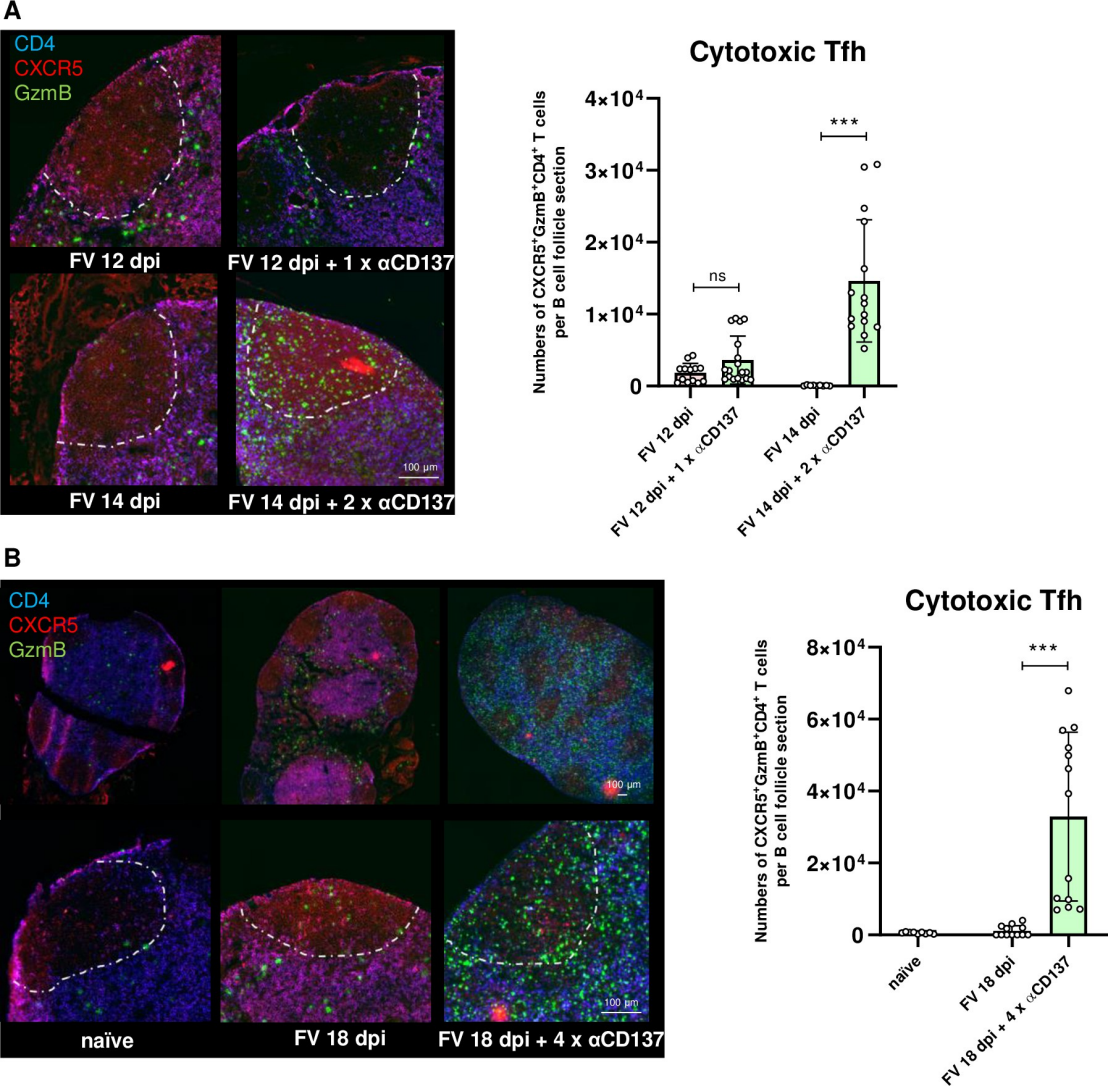
Cytotoxic Tfh cells, induced by αCD137 therapy, present in B cell follicles. CB6F1 mice were infected with FV, treated once with αCD137 and sacrificed on day 12 pi, treated twice with αCD137 and sacrificed on day 14 pi, or four times with αCD137 and sacrificed on day 18 pi. One inguinal lymph node from every experimental mouse was extracted on the day of the experiment. Samples were frozen in liquid nitrogen, processed to cryosections, fixed, and stained with fluorescently-labeled antibodies. Multichannel images were acquired with Axio Scan.Z1 using Plan-Apochromat 20x/0.8 M27 objective and Zen blue software. Representative images of B cell follicles are shown. (A) B cell follicles after one (upper row) or two (lower row) injections of αCD137 antibody compared to untreated FV-infected animals (left). (B) Low magnification images of the entire lymph node (upper row) and higher magnifications of B cell follicles (lower row) after four injections of αCD137 antibody compared to naïve (left) and untreated FV-infected animals (middle). The graph shows the numbers of cytotoxic Tfh cells per B cell follicle section. Dots indicate values of single B cell follicle. Median of the groups ± SD is given; ns–not significant, ***p < 0.001, Mann-Whitney test. Green signal represents GzmB^+^ cells, blue–CD4^+^ cells, red–CXCR5^+^ cells. Scale bar = 100 μm. The areas of B cell follicles within lymph nodes are indicated with the dotted line.

As a next step, inguinal lymph nodes collected after one, two or four αCD137 injections, as well as lymph nodes from naïve and FV-infected control mice, were snap frozen in liquid nitrogen and subjected to immunofluorescent microscopy. To visualize induced cytotoxic Tfh cells, we stained lymph node sections with antibodies against CD4, CXCR5, and GzmB. Cytotoxic Tfh cells defined as triple positive cells were identified within the B cell follicles (Fig 2). The absolute numbers of such cytotoxic Tfh cells within the follicles were significantly increased already after two injections of the antibody (Fig 2A). However, due to the additive therapeutic effect of further injections the most striking impact on absolute numbers of cytotoxic Tfh cells was found after 4 antibody injections (compare Fig 2A and 2B). Hence, cytotoxic Tfh indeed present in lymph node B cell follicles after immunotherapy and co-localize with FV reservoir cells.

### αCD137 therapy reduces viral loads in many B cell follicles to undetectable levels

**Fig 3:**
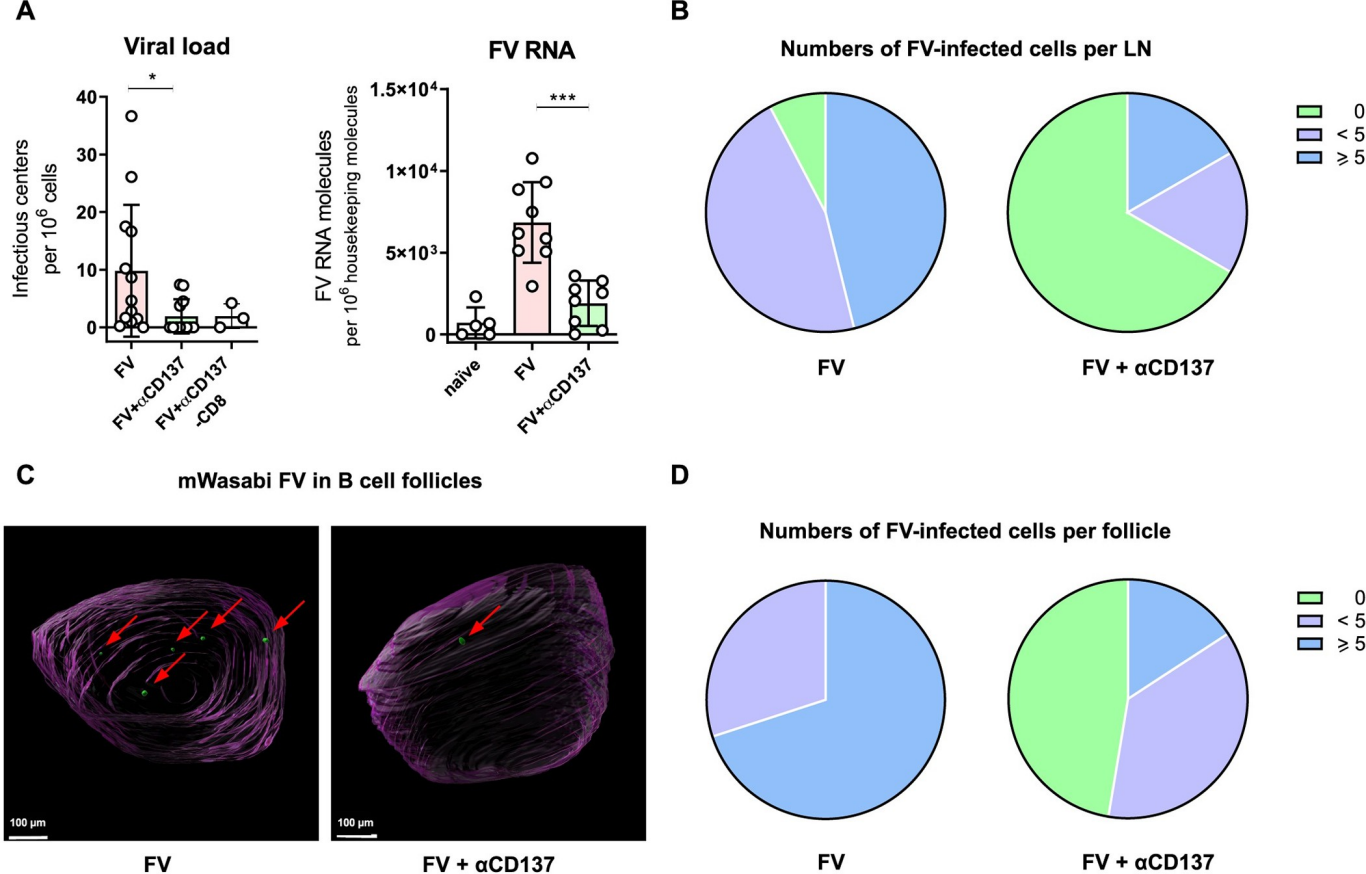
αCD137 therapy induces viral load reduction in lymph nodes and B cell follicles. CB6F1 mice were infected with mWasabi-expressing FV, αCD137-treated from 10–16 dpi, and sacrificed on day 18 pi. (A) Viral loads in lymph nodes presented as infectious centers per 10^6^ cells (IC assay) or viral RNA copies per 10^6^ housekeeping molecules (RT-qPCR). Dots indicate results for single mice ± SD; *p < 0.05, ***p < 0.001, Mann-Whitney test. (B) The proportion of FV-infected cells per lymph node (LN) for the categories: no detectable FV-infected cell, less than five FV-infected cells, five or more FV-infected cells based on IC assay. (C) Representative images of B cell follicles, obtained via two-photon microscopy. Green dots represent FV-infected mWasabi^+^ cells. Scale bar = 100 μm. (D) The proportion of FV-infected cells per B cell follicle in each category: no detectable FV, less than five FV-infected cells, five or more FV-infected cells based on two-photon microscopy. Four follicles per lymph node of five mice per group were analyzed.

The expansion of Tfh with a cytotoxic phenotype after αCD137 agonist therapy suggested that they might be able to kill infected cells and reduce virus in the reservoir. To determine viral loads after therapy in lymph nodes (pooled inguinal, axillary, brachial lymph nodes from both left and right side of the mouse and four superficial cervical lymph nodes), we performed infectious center (IC) assay and qRT-PCR (Fig 3A). Antibody treatment resulted in a significant reduction of FV levels (IC and viral RNA levels) compared to FV-infected untreated animals. Furthermore, the fraction of virus-free lymph nodes measured by the IC assay was five times higher in the treated group compared to the untreated control (Fig 3B). The elimination of infected cells from lymph nodes was independent of CD8^+^ T cells because an experimental depletion of CD8^+^ cells prior to therapy did not change the results (Fig 3A).

The reduction in viral loads in total lymph nodes upon therapy was interesting, but our main interest was to study the reservoir in B cell follicles. We therefore visualized single B cell follicles of lymph nodes by utilizing light sheet two-photon microscopy. Visualization of individual FV-infected cells was feasible due to the use of the recombinant mWasabi-FV. Lymph nodes of mWasabi-FV-infected αCD137-treated or untreated mice were harvested and examined under the microscope (Fig 3C and S1 Video). B cell follicles of mice receiving the therapy contained no or only very low numbers of mWasabi^+^ FV-infected cells. Almost half of the investigated B cell follicles were virus-free in the treated group, whereas none of the analyzed B cell follicle from untreated mice was virus-free (Fig 3D).

### FV-infected follicular lymphocytes are possible targets for cytotoxic CD4^+^ T cells

The T cell receptor of CD4^+^ T cells recognizes antigen epitopes that are bound to MHC class II molecules on antigen-presenting cells or target cells. However, MHC class II is not expressed on all body cells, so the question arises if cytotoxic Tfh can recognize retroviruses-infected follicular cells. In FV infection follicular T and B cells form the viral reservoir and it is well established that B cells express MHC class II. Not surprisingly, in our current experiments more than 95% mWasabi^+^ B cells were MHC class II positive (S2A Fig). In addition, we also found that more than half of the FV-infected Tfh in lymph nodes expressed MHC class II (S2B Fig). That is very similar to the situation in HIV, in which HLA-DR is even used as marker to find the viral reservoir Tfh cells in the lymph nodes of infected individuals [33]. Thus, retrovirus-infected cells in B cell follicles, either T or B cells, are suitable targets for cytotoxic CD4^+^ Tfh cells.

### aCD137 therapy delays viral rebound in lymph nodes upon ART interruption

**Fig 4:**
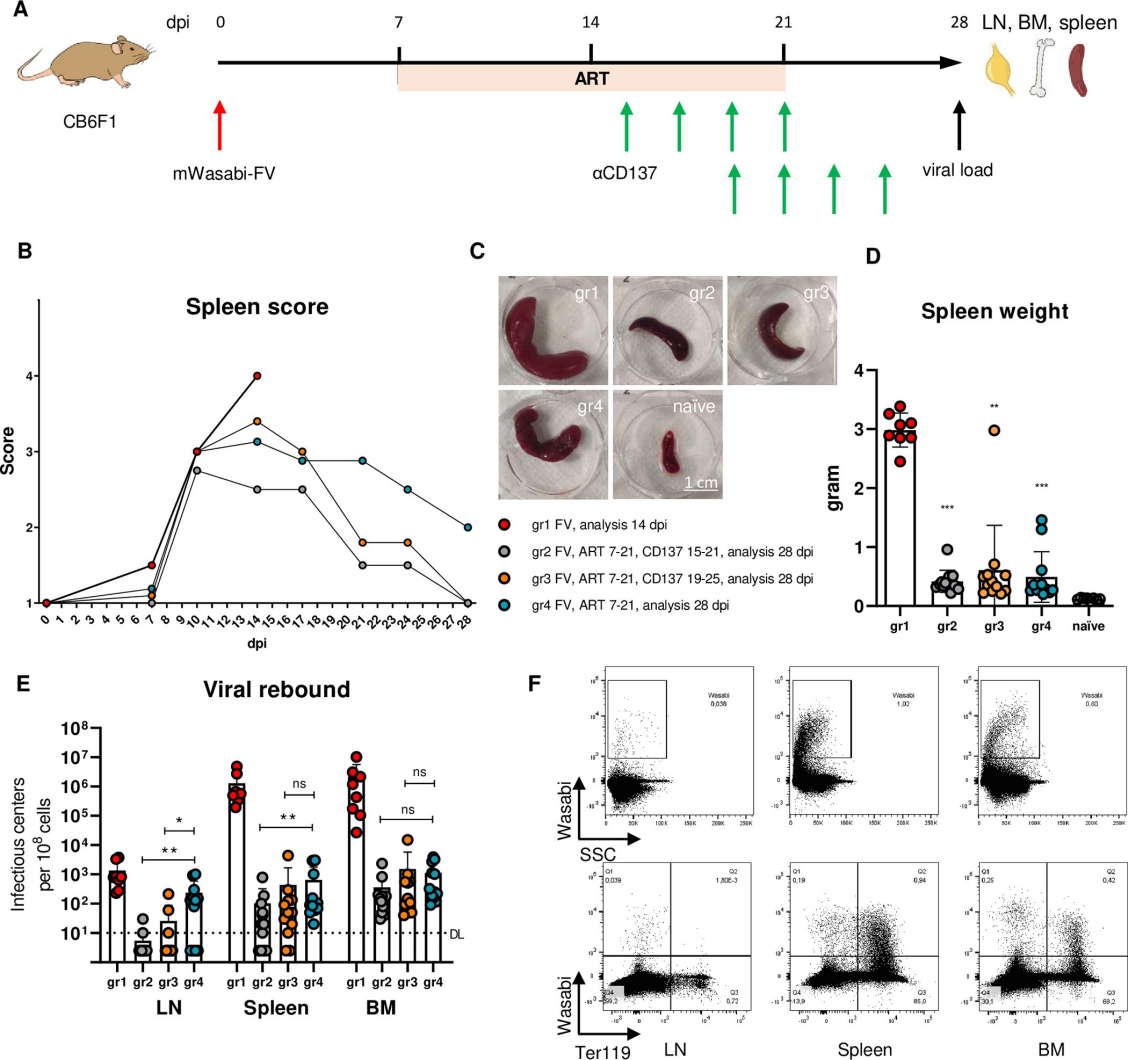
αCD137 therapy prevents viral rebound in lymph nodes upon ART termination. CB6F1 mice were infected with mWasabi FV, ART-treated from 7–21 dpi and two groups were in addition αCD137-treated from 15–21 dpi (gr2) or 19–25 dpi (gr3). The control groups were mice that did not receive αCD137 antibody (gr4) or left untreated (gr1) (A). (B) Spleen scores assigned with a semi-quantitative palpation method [81]. Each dot represents the mean score of the group. (C) Representative images of spleens from one mouse of each group. Scale bar = 1 cm. (D) Spleen weights following sacrifice of the animals at 14 dpi (group 1) or 28 dpi (groups 2–4). Dots indicate values from single mice ± SD; **p < 0.01, ***p < 0.001, one-way ANOVA. (E) Viral loads in lymph nodes (LN), spleen, and bone marrow (BM) presented as infectious centers per 10^8^ cells. Dots indicate values from individual mice. Median of the groups ± SD is given; dotted line DL–detection limit, ns–not significant, *p < 0.05, **p < 0.01, one-way ANOVA. (F) Representative dot plots of lymph node (LN), spleen, and bone marrow (BM) cells. mWasabi^+^ FV-infected cells versus side scatter (upper row) or versus Ter119^+^ erythroid precursor cells (lower row).

To control human HIV infection, ART is available in the clinic. ART is a combination of different drug classes, such as reverse transcriptase inhibitors, protease inhibitors, or integrase inhibitors. Although ART efficiently suppresses HIV replication in most patients, it does not eliminate the viral reservoir, which is the source for viral rebound upon treatment interruption. To model this human situation, we treated FV-infected mice with an ART that efficiently suppressed FV replication (S3 Fig). The ART consisted of Azidothymidine, Raltegravir, and Darunavir and was administered to the mice with chow. ART from day 3–14 pi significantly reduced FV loads and extended therapy from day 0–21 dpi suppressed viral loads under the limit of detection (S3B Fig). In the αCD137 treatment experiment ART was started at 7 dpi (Fig 4A), to allow for seeding of the viral reservoir, similar to humans who very rarely start ART immediately after infection. ART was supplemented in two groups with four αCD137 injections started either at 15 dpi or 19 dpi. ART-containing chow was replaced by regular food one week before the termination of the experiment to allow for viral rebound from the reservoir. FV-infected untreated control animals were sacrificed on day 14 pi to prevent the development of severe disease (Fig 4B and 4C).

One of the prominent symptoms of FV-induced disease is splenomegaly [34]. To demonstrate that ART improved FV pathology, just like ART does in HIV infection, we analyzed the spleen score during the experiment and the spleen weight upon termination of the experiment. The spleen score was accessed through gentle palpation of the left side of the mouse’s abdomen and assigned score on a semi-quantitative scale from one to four. All three ART regimens supplemented or not with αCD137 therapy significantly improved the course of infection by reducing the spleen score and spleen weight (Fig 4B–4D). However, the critical question was, whether the αCD137 antibody therapy restricted the viral rebound upon ART interruption, therefore we performed an IC assay with cells from lymph nodes, spleen, and bone marrow (Fig 4E). In lymph nodes, the reservoir site that we were most interested in, viral loads rebounded to levels of untreated mice upon ART interruption. In contrast, additional treatment with αCD137 antibodies significantly reduced viral loads and this therapeutic effect on viral rebound was found with earlier (15 dpi) and later (19 dpi) onset of antibody treatment. In antibody treated mice, lymph node viral loads were below the detection limit in many animals, suggesting that immunotherapy delayed FV rebound.

Cytotoxicity of CD4^+^ Tfh cells was assessed seven days following the last injection of the αCD137 antibody. At this time point, the frequencies of CD4^+^ Tfh cells expressing the cytotoxic markers started to decline (S4 Fig). Thus, the effects of immunotherapy were not sustained. We believe that this might be a therapeutic advantage, since a permanent induction of cytotoxic Tfh is not desirable.

We also investigated other organs, because FV efficiently infects the bone marrow and spleen, but those organs have a different anatomic architecture than lymph nodes. In both organs, viral loads were medium high after ART interruption, but additional αCD137 antibody therapy had no effect on spleen viral loads except for the 15-dpi treatment start (group 2). This suggests that the different organ architecture and maybe another target cell reservoir for FV infection might influence the therapeutic potential of αCD137 antibodies. To address this, we utilized mWasabi-FV for detecting FV target cell populations in the spleen, lymph nodes, and bone marrow of FV-infected untreated mice (Fig 4F). As expected, Wasabi^+^ cells were detected in all FV-infected organs and an important target cell population of FV was Ter119^+^ erythroid precursor cells. Ter119^+^ erythroblasts were reported to be essential for FV pathogenesis [35] through induction of proliferation and subsequent infection of this cell population. These erythroid precursor cells form the main viral reservoir in the spleen and bone marrow (Fig 4F), which explains why the Tfh-targeting therapy did not efficiently reduce viral loads here. However, these FV target cells did not play a significant role in lymph nodes, but here the viral reservoir of follicular T and B cells could be attacked by the therapy-induced cytotoxic Tfh.

### αCD137 therapy does not damage B cell follicles or abolish humoral immune responses

**Fig 5:**
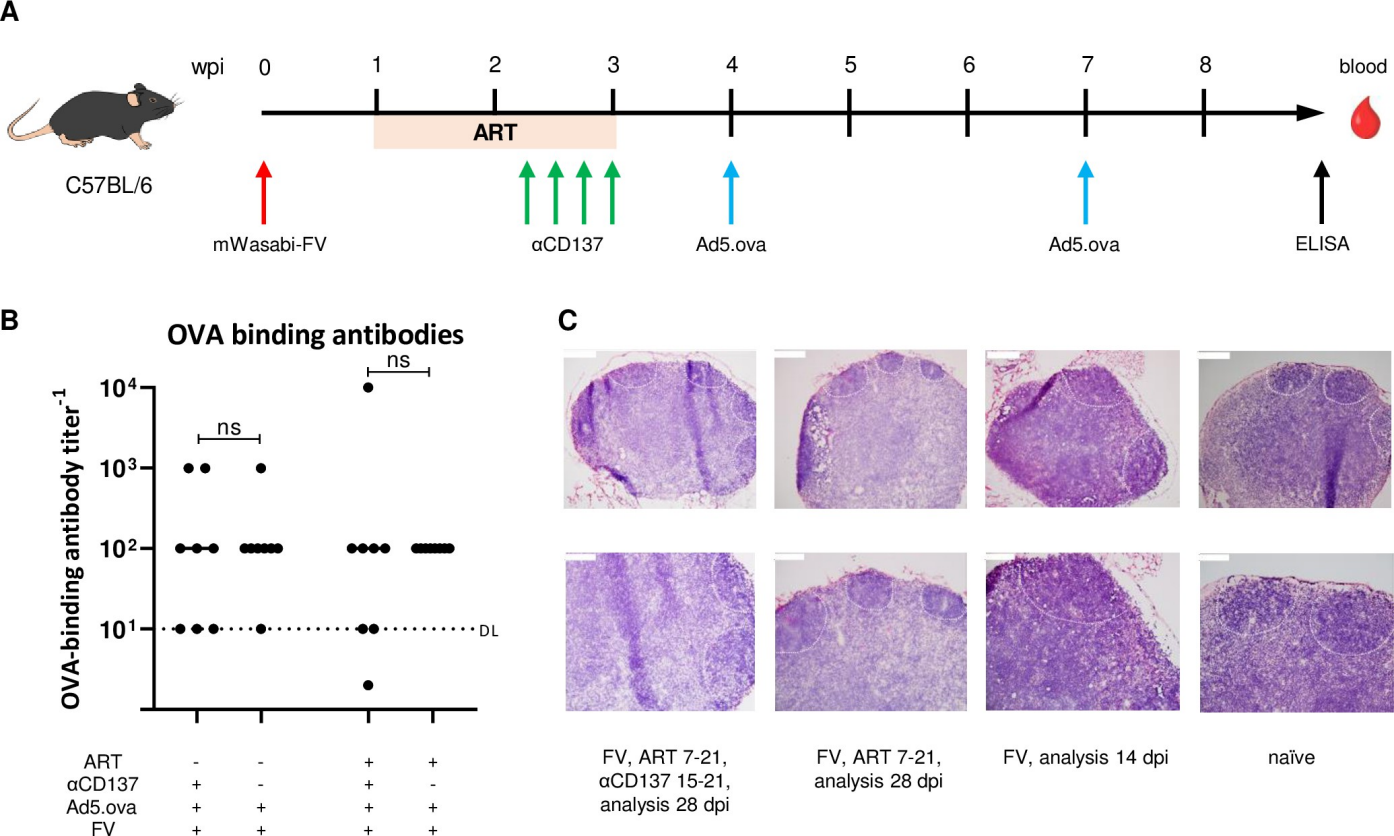
αCD137 therapy does not diminish humoral immune responses or damage lymph node structure. C57BL/6 mice were infected with mWasabi-FV, ART-treated from 7–21 dpi, αCD137-treated from 15–21 dpi and subsequently Ad5.ova-vaccinated on 28 and 49 dpi. Blood was collected on day 63 pi before mice were sacrificed (A). OVA-binding antibody titers were determined by ELISA (B). Dots indicate values from individual mice for highest positive plasma dilution; ns–not significant. Dotted line DL–detection limit. (C) Representative images of hematoxylin and eosin-stained lymph node sections. Lymph nodes were acquired from mice described in experimental setup Fig 4A. Low magnification images (upper row) of the entire lymph node. High magnification images (lower row) show densely packed lymphocytes in the B cell follicles in the cortex region and loose lymphoid tissue of the medulla. Scale bar = 200 μm (upper row) and 100 μm (lower row).

Tfh are not cytotoxic under physiological circumstances, however the αCD137 antibody therapy initiates their cytotoxic program. An obvious concern is that this might induce damage in B cell follicles and interfere with antibody responses. To address this, we treated FV-infected mice with ART and αCD137 and after therapy was completed, mice were immunized with the adenovirus-based vaccine vector Ad5.ova. This vector encodes the chicken egg protein ovalbumin, which is known to induce strong antibody and T cell responses in mice. For this experiment we used C57BL/6 mice because they develop chronic FV infection and do not develop FV-induced disease which would limit the possible experimental time span (Fig 5A).

Two immunizations were shown to be required for the induction of strong antibody responses with adenoviral vectors [36], therefore two Ad5.ova inoculations were performed on day 28 and 49 pi. Blood samples were acquired two weeks after the second immunization to determine humoral immune responses. Following the second immunization, ova-binding antibodies were detectable in all mice except one, and αCD137 therapy did not impair humoral immune responses when applied as monotherapy or in combination with ART (Fig 5B).

Moreover, inguinal lymph nodes collected from CB6F1 mice treated with the combination therapy as described in Fig 4A, were snap-frozen, stained with hematoxylin and eosin, and microscopically analyzed for their typical lymph node morphology (Fig 5C). The evaluation of lymph nodes acquired from naïve, FV-infected untreated, or treated animals showed clearly visible B cell follicles and T cells zones and no alteration of the normal histology in any of the mice.

Thus, our current study indicates that αCD137 immunotherapy induces the cytotoxic program in Tfh, which subsequently enter B cell follicles in lymph nodes and reduce the retroviral reservoir without disturbing the architecture or antibody responses.

## Discussion

The recent success of immunotherapies in the treatment of malignancies brought the idea to broaden their application to the field of infectious diseases. Host-directed antiviral immunotherapies have been described for different viruses including influenza [37], HIV [38], and most recently SARS-CoV-2 [39]. Although agonistic antibodies binding the TNF receptor family member CD137 are under investigation in anti-tumor therapy, little attention has been paid to their role in viral infections. In the current study, we show that αCD137 therapy induces the cytotoxic program in Tfh cells, which are licensed to enter B cell follicles and can therefore contribute to the elimination of the Friend retrovirus reservoir. Interestingly, almost half of the investigated B cell follicles were virus-free upon this therapy. Moreover, the administration of the agonist together with ART reduced viral rebound following therapy interruption. Despite the robust cytotoxicity of induced Tfh cells, the structure and function of lymph nodes and B cell follicles were not affected.

The potential of CD137 agonist to abolish cancer progression in humans was first acknowledged in 2008 when Urelumab, the CD137-targeting human monoclonal antibody, entered clinical trials [40]. Preliminary anti-tumor results were impressive, but studies had to be terminated due to unexpected hepatotoxicity [15]. Another anti-human CD137 agonist Utomilumab showed a better safety profile [41], however demonstrated only moderate anti-tumor activity [42]. A novel strategy to overcome the hepatotoxicity is the development of bi-, tertiary, or even tetraspecific antibodies targeting CD137 simultaneously with other immune checkpoints, for instance PD-L1, HER2, or EGFR [43]. Moreover, a novel anti-CD137 antibody was developed that binds to a different CD137 epitope and therefore does not lead to systemic toxicity [44]. This antibody was successfully tested against colorectal cancer in humanized mice and cynomolgus monkeys. Hence, despite persisting safety issues, anti-human CD137 remains a highly interesting target for immunotherapy.

Cytotoxic CD4^+^ T cells were described to compensate for the CD8^+^ killing capacity in different viral infections under certain circumstances, especially in chronic infections when CD8^+^ T cell exhaustion develops [45, 46]. However, cytotoxic Tfh seem to be very rare cells even in infectious diseases in which the virus can infect cells in lymph nodes or even in B cell follicles. This is an important feature of immune escape and reservoir formation in some viral infections [47, 48]. Here, we induced cytotoxicity of Tfh via αCD137 antibody administration. In humans activated CD4^+^ T cells are preferential targets of HIV [49], so their stimulation *in vivo* appears controversial. It has been shown that activation of CD4^+^ T cells by a prototype HIV vaccine results in enhanced viral replication in CD4^+^ T cells instead of vaccine protection [50]. Moreover, studies highlight the essential role of CD4^+^ Tfh in HIV reservoir formation and maintenance [51, 52], so their activation might enhance infection. On the other hand, the beneficial effect of cytotoxic CD4^+^ T cells in virus control is supported by several observations. HIV-specific cytotoxic CD4^+^ T cells were detected in high frequencies in elite controllers [53] and were associated with lower viral set points [54]. Moreover, HIV and SIV Gag-specific CD4^+^ T cells exert selective pressure on the virus that leads to the emergence of viral escape mutations [55, 56]. Therefore, despite the fact that CD4+ T cells are targets of HIV, uninfected CD4^+^ T cells, including Tfh cells in viral reservoirs, can become antiviral effectors during chronic infection.

Tfh expressed high frequencies of the cytotoxic markers Eomes and GzmB after therapy, which was virtually absent during FV infection only. The appearance of the cytotoxic Tfh correlated with a significant reduction of the FV reservoir in lymph nodes. However, CD8^+^ T cells were also described as being consumers of αCD137 antibodies [43]. This essential feature was utilized in the development of novel oncological drugs where CD137 agonists showed promising anti-cancer effects mainly due to the cytotoxic effector function of CD8^+^ T cells [57, 58]. Here we need to mention a subset of CXCR5^+^ follicular CD8^+^ T cells that received increasing attention in the past few years. Despite the fact that it was first identified more than ten years ago [59], only recently the therapeutic potential of this subset underwent intensive investigation [60]. For instance, PD-1/PDL-1 blocking immunotherapy contributed to a viral load reduction in the lymphocytic choriomeningitis virus mouse model via the expansion of these follicular CD8^+^ T cells [61, 62]. Additionally, they were found in the germinal centers of individuals who spontaneously control HIV infection [63]. Since these cells can potentially enter follicles in lymph nodes due to their CXCR5 expression, they could theoretically contribute to viral reservoir elimination. So, were cytotoxic CD8^+^ T cells instead of Tfh responsible for the therapeutic effect on αCD137 therapy in the FV model? This was very likely not the case, because the follicular CD8^+^ T cell subset did not expand after αCD137 treatment and therefore did not correlate with therapy outcome.

Reduction or elimination of the viral reservoir is an important aim for HIV cure strategies. Complex antiretroviral drug combinations became the golden standard in HIV care and led to a major therapeutic success. However, despite the fact that the life expectancy has increased for HIV-infected patients on adequate ART regimens, individuals with HIV have higher risks of cardiovascular diseases compared to uninfected control cohorts [64] [65]. Also, antiretroviral medications may cause metabolic disorders such as insulin resistance or elevated cholesterol levels, which in turn promote the development of other diseases [66]. Current ART has very little to no influence on the HIV reservoir size and the reservoir very likely negatively influences some of the health issues of people living with HIV (PLWH) on optimal ART experience. Thus, novel strategies and drugs are required to target the retroviral reservoir and to improve the healthcare management of PLWH.

We demonstrate here that administration of the agonist to CD137 reduced viral rebound in lymph nodes after ART discontinuation. Moreover, a substantial number of αCD137-treated mice presented with virus-free lymph nodes if antibody treatment was supplemented after the beginning of ART. In the FV model, however, this does not result in viral elimination from the host, because also the spleen and bone marrow, where Ter119^+^ erythroid precursor cells represented the bulk of infected targets, are infected. Virus reactivation from these organs cannot be controlled by cytotoxic Tfh. This cell type does not play a role in HIV-infected individuals, so the FV model does not fully reflect the human situation in this case. However, the effective viral control in lymph nodes indicates the potential of the current therapy approach to target the retroviral reservoir.

Interestingly, cytotoxic Tfh cells were found in the B cell follicles that are described to harbor retroviral reservoirs. Several studies reported that the induction of cytotoxicity in CD4^+^ T cells can be detrimental since such cells contribute to immunopathology [67]. Additionally, several immunotherapies are facing the risk of immunopathology following treatment, so called immune-related adverse events. In fact, we also showed that combined immunotherapies can induce lethal immunopathology in the FV model [68]. Moreover, it was reported that some immunotherapies, such as TNF inhibitors, impair vaccine responses since TNF functions include germinal center formation and immunoglobulin synthesis induction [69–71]. In a mouse model, NK cells were reported to reduce Tfh and germinal center B cell frequencies in a perforin-dependent manner, negatively affecting germinal center formation [72]. Therefore, there is a potential danger of damaging B cell follicles and subsequent humoral immune responses by immunotherapies that activate cytotoxic cells.

However, no difference was found in antibody levels to ovalbumin antigen in the αCD137-treated group compared to untreated controls and no anatomic damage was visible. One obvious explanation for this positive finding is, that the number of infected reservoir cells in lymph nodes is rather low during persistent FV infection [6], thus killing of these cells has no physiological consequences. This is an obvious similarity to HIV where numbers of infected cells in lymph nodes are also rather low during chronic infection especially under ART [73]. Thus, our αCD137 therapy approach seems to be safe for targeting the retroviral reservoir in lymph nodes.

In conclusion, we showed that the treatment of FV-infected mice with αCD137 agonistic antibody resulted in the induction of cytotoxic Tfh CD4^+^ T cells, which resulted in viral load reduction and initiated at least partial clearance of the viral reservoir. The persistence of the latent reservoir is the main obstacle to cure HIV infection [74]. To combat this challenge, “shock and kill” strategies were intensively studied in the last decades. Important problems in targeting the reservoir in retroviral infections are not only its location in immune-privileged sides, like germinal centers, but also that a part of the reservoir cells is latently infected and cannot be recognized by cytotoxic killers. Thus, the shock approach is based on the administration of latency-reversing agents (LRAs) that make infected cells visible as targets for elimination. It has been shown that LRAs alone can activate HIV transcription, but they are ineffective at killing infected cells [75]. We therefore suggest to combine αCD137 antibody therapy with LRAs as an even more effective combination therapy for future research on HIV reservoir elimination and cure or functional cure studies.

## Materials and methods

### Ethics statement

All mouse experiments were carried out with permission from the LANUV NRW (Landesamt für Natur, Umwelt und Verbraucherschutz Nordrhein-Westfalen, North Rhine-Westphalia State Office for Nature, Environment and Consumer Protection), in compliance with national law, and in accordance with institutional guidelines of the University Hospital Essen, Germany. Written informed consent from the participants for the experiments with PBMCs was obtained. The blood collection was approved by the Ethics Committee (No.:11–4715) of the University of Duisburg-Essen.

### Mice and virus

Female BALB/c, CB6F1, and C57BL/6 mice were purchased from Charles River (Erkrath, Germany). Fluorescently labelled FV containing mWasabi-modified Friend murine leukemia virus has been described before [6]. Different strains of mice were infected with different doses of mWasabi-FV in PBS (BALB/C with 500 SFFU, CB6F1 1500 SFFU, and C57BL/6 20 000 SFFU respectively) by intravenous injection of total volume of 100 μl.

### αCD137 antibody treatment and ART

The anti-CD137 (clone LOB 12.3) antibody was purchased from BioXCell (Lebanon, NH, USA). Antibody was administered via intraperitoneal injection of 100 μg/mouse diluted in PBS for every application. CB6F1 mice received one, two or four injections starting at day 10 pi every second day for monotherapy. Administered in combination with ART, antibody was provided to CB6F1 mice four times at the same dose starting at day 15 or 19 pi, and to C57BL/6 mice at day 15 pi.

ART was provided with chow and consisted of 666 mg/kg Retrovir, 2660 mg/kg Darunavir, and 1600 mg/kg Raltegravir (Ssniff Spezialdiäten, Soest, Germany). ART chow was provided to BALB/c mice days 3 to 14 pi, or days 0 to 28 pi; and to CB6F1 as well as C57BL/6 mice days 7 to 21 pi.

### Ad5.ova vaccination

The adenovirus type 5 based, chicken egg ovalbumin encoding vector Ad5.ova has been described before [76]. C57BL/6 mice were immunized with 1 × 10^9^ viral particles of Ad5.ova diluted in 30 μl of PBS by intramuscular injection into the Musculus gastrocnemius. Prime-boost immunizations were performed in a 3-week interval at days 28 and 49 pi.

### RNA isolation

To estimate FV infection levels, RNA was isolated from ten pooled lymph nodes from every experimental mouse (two inguinal, two brachial, two axillary, four superficial cervical) using innuPREP RNA Mini Kit 2.0 (Analytik Jena, Jena, Germany) following the manufacturers protocol. Eluted RNA was stored at -80°C.

### Reverse transcriptase quantitative PCR

RT-qPCR to estimate FV infection levels has been described previously [77]. mRNA molecules were quantified using Rotor-Gene Q cycler (Qiagen, Hilden, Germany).

### Infectious center (IC) assay

Single-cell suspensions of lymph nodes, spleens, and bone marrow were plated onto to FV permissive *Mus dunni* cells in different dilutions. Upon reaching confluence, immunocytochemical staining using the hybridoma-derived antibody 720 was performed to identify infected cells as previously described [78].

### Fluorescence microscopy and histological examination

One inguinal lymph node from every experimental mouse was extracted on the day of the experiment. Samples were loaded into Tissue-Tek O.C.T. Compound (Sakura Finetek, Japan), frozen in liquid nitrogen and stored at -80°C until use. Subsequently, lymph nodes were processed to 5 μm cryosections. For fluorescent staining, sections were fixed with BD Cytofix/Cytoperm (BD Biosciences, Franklin Lakes, NJ, USA) and stained with rat anti-mouse CXCR5 (614641, Bio-Techne, Minneapolis, MN, USA) for 90 min at room temperature and polyconal goat anti-mouse Granzyme B (Bio-Techne) overnight at 4°C. Afterward, sections were stained with secondary donkey anti-rat, donkey anti-goat (both Dianova, Hamburg, Germany) antibodies for 45 min at room temperature followed by anti-CD4 (RM4-5, BioLegend, San Diego, USA) for 90 min at room temperature. DAPI was used for nucleus counter staining. After staining, multichannel images were acquired with Axio Scan.Z1 using Plan-Apochromat 20x/0.8 M27 objective and Zen blue software (both Carl Zeiss Microscopy, Oberkochen, Germany) at the Imaging Facility Essen (IMCES). Definiens Tissue Studio image analysis software (Definiens Inc., Carlsbad, CA, USA) was used to characterize the area of B cell follicles within the lymph nodes and quantify cells of interest. Cytotoxic Tfh cells were defined as CD4^+^ CXCR5^+^ Granzyme B^+^ cells.

For histological examination, samples were fixed with cold acetone and stained with hematoxylin and eosin. Visualization was performed using Olympus BX51 epifluorescence microscope (Shinjuku, Japan). Lymph node tissues were analyzed for the presence of typical morphological formations (B cell follicle, T cell zone).

### Two-photon microscopy

One inguinal lymph node from every experimental mouse was removed upon transcardial perfusion of sacrificed mice with EDTA/PBS followed by 4% formaldehyde in PBS (pH 7.4). Next, lymph nodes were incubated with shaking at 4°C in 4% formaldehyde in PBS for 4 h, washed with PBS for 2 h and incubated with CUBIC-1 reagent [79] for 5 days with gentle shaking at 37°C. Optically cleared lymph nodes were analyzed by two-photon microscopy as described before [6]. mWasabi-FV-infected cells, autofluorescence and collagen structures were visualized and analyzed with Imaris 9.2.1 software (Bitplane AG, Zurich, Switzerland).

### Infection of PBMCs with HIV and αCD137-co-culture

Blood samples from healthy donors were donated at the University Hospital Essen. PBMCs from these blood samples were isolated by density gradient centrifugation as described elsewhere [80]. One day before infection, PBMCs were kept in culture medium (RPMI + 10% FCS + 1% P/S, + 1% HEPES) containing 1 μg/ml PHA and 10 ng/ml IL2 (Miltenyi Biotec, Bergisch Gladbach, Germany). Then, cells were infected with X4-tropic HIV-1_NL4-3_IRES_eGFP_ at a MOI of 0.5 via spinoculation at 1,500 x g for 2h at room temperature. On day 5 pi, 10 μg/ml agonist anti-human CD137 (BPS Biosciences, San Diego, USA) was added. On day 6 pi, cells were collected for CD107a degranulation assay.

### Flow cytometry and CD107a degranulation assay

Cells were washed, placed to a 96 well plate and stained with fluorescently-labeled anti-mouse antibodies for CD3 (17A2 or 145-2C11, BD; or 17A2, BioLegend), CD4 (GK1.5, BD), CD8 (53–6.7, BD, BioLegend or Thermo Fisher Scientific, Waltham, USA), CD19 (6D5, BioLegend), CXCR5 (L138D7, BioLegend), PD1 (RMP1-30, BioLegend), biotin Ter119 (TER-119, BD, coupled to BUV737 Streptavidin, BD), I-A/I-E (M5/114.15.2, BioLegend), MHC class-II tetramer loaded with I-A b-restricted FV-specific CD4^+^ T-cell epitope (previously described [11]), and anti-human antibodies for CD107a degranulation assay to CD3 (OKT3, BioLegend), CD4 (OKT4, BioLegend), CD8 (BW135/80, Miltenyi Biotec), CD19 (HIB19, BioLegend), CD107α (LAMP-1, BioLegend). Cells were stained with fixable viability dye (Thermo Fisher Scientific or Zombie UV, BioLegend) for the exclusion of dead cells from subsequent analysis. The MHC class II tetramer staining was carried out in the dark for 2 h at 37° C. The viability and surface stains were carried out in the dark for 20 minutes at room temperature.

Following, mouse cells were fixed for 2 h with eBioscience Foxp3/Transcription Factor Staining Buffer Set (Thermo Fisher Scientific) and stained intracellularly for 1 h with antibodies for Eomesodermin (Dan11mag, BioLegend), Granzyme B (GB11, BD), and Foxp3 (FJK-16s, Thermo Fisher Scientific). PBMCs were fixed with PFA-containing permeabilization buffer for 2 h, washed and measured. Mouse samples and PBMCs were aquired with FACSymphony flow cytometer (BD). Data analysis were performed using FlowJo software (BD).

### Ovalbumin ELISA

For the detection of ova-binding antibodies, ELISA 96-well plate (Nunc, Roskilde, Denmark) was coated with ova protein (5 μg/ml; Sigma-Aldrich, Munich, Germany). Subsequently, plate was blocked with 10% FCS in PBS and incubated with serial dilutions of mouse plasma. Antibodies were detected with anti-mouse immunoglobulin antibody (polyclonal rabbit anti-mouse HRP-coupled antibody, Dako GmbH, Germany) and tetramethylbenzidine (Dako GmbH, Germany). Sample dilutions were considered positive if the absorption was two-fold higher than that in naïve mice.

### Statistics

Statistical analysis and visualization of the results were performed using GraphPad Prism software (GraphPad, San Diego, CA, USA). The Mann-Whitney test was used to determine the statistical differences between two groups. The nonparametric Kruskal-Wallis one-way analysis of variance (ANOVA) test was used to test analyses involving multiple groups.

## Supporting information

S1 Fig αCD137 antibody induces CD107a expression in human CD4^+^ T cells. Human PBMCs isolated from healthy donors were infected with X4-tropic HIV-_1NL4-3_IRES_eGFP_. Agonist anti-human CD137 was added on day 5 pi for 24 h. Expression of CD107a degranulation marker was assessed using flow cytometry. Data is shown from two independent experiments. Dots indicate individual donors. Median of the groups ± SD is given, *p < 0.05, Mann-Whitney test.
(PDF)

S2 Fig FV-infected cells express MHC class II and can be targets of CD4-mediated killing. Representative dot plots of mWasabi-labeled FV-infected B cells (A) and Tfh cells (B) from lymph nodes were stained for MHC II expression on their surface.
(PDF)

S3 Fig ART suppresses FV infection in mice. BALB/c mice were infected with FV, left untreated (gr1), ART-treated from 3–14 dpi (gr2), or ART-treated from 0–28 dpi (gr3) and sacrificed on 14 dpi (gr1, 2) or 28 dpi (gr3) (A). Single-cell suspensions from lymph nodes (LN), bone marrows (BM), and spleens were isolated and used for assessment of viral loads with an infectious center assay (B). Dots indicate values of individual mice. Median of the groups ± SD is given; dotted line DL–detection limit, **p < 0.01, ***p < 0.001, one-way ANOVA.
(PDF)

S4 Fig Expression of cytotoxic markers of CD4^+^ Tfh cells declines over time after the αCD137 injection. CB6F1 mice were infected with mWasabi-expressing FV, treated with αCD137 (B and C) and/or ART (A and C). Dot plots from representative mice indicate the expression of cytotoxic molecules (Eomes and GzmB) by CXCR5^+^ PD1^+^ CD4^+^ T cells.
(PDF)

S1 Video αCD137 therapy induces viral load reduction in B cell follicles. CB6F1 mice were infected with mWasabi-expressing FV, and sacrificed on day 18 pi. Representative video of B cell follicle, obtained via two-photon microscopy. Green dots represent FV-infected mWasabi^+^ cells.
(MP4)

S2 Video αCD137 therapy induces viral load reduction in B cell follicles. CB6F1 mice were infected with mWasabi-expressing FV, αCD137-treated from 10–16 dpi, and sacrificed on day 18 pi. Representative video of B cell follicle, obtained via two-photon microscopy. Green dot represents FV-infected mWasabi^+^ cell.
(MP4)
