# Supplementary material for: Immunotherapy-induced cytotoxic T follicular helper cells reduce numbers of retrovirus-infected reservoir cells in B cell follicles
Source: PLoS Pathog. 2023 Oct 26;19(10):e1011725. doi: 10.1371/journal.ppat.1011725 (PMC10602292; doi:10.1371/journal.ppat.1011725)
Supplement: S1 Fig — Human PBMCs isolated from healthy donors were infected with X4-tropic HIV-1NL4-3_IRES_eGFP. Agonist anti-human CD137 was added on day 5 pi for 24 h. Expression of CD107a degranulation marker was assessed using flow cytometry. Data is shown from two independent experiments. Dots indicate individual donors. Median of the groups ± SD is given, *p < 0.05, Mann-Whitney test. (PDF) [file ppat.1011725.s001.pdf]

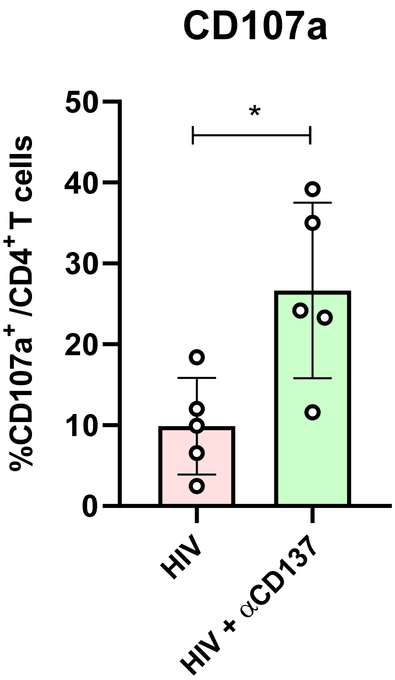

S1 Fig. αCD137 antibody induces CD107a expression in human CD4⁺ T cells.

Human PBMCs isolated from healthy donors were infected with X4-tropic HIV-$_{1NL4-3\_IRES\_eGFP}$. Agonist anti-human CD137 was added on day 5 pi for 24 h. Expression of CD107a degranulation marker was assessed using flow cytometry. Data is shown from two independent experiments. Dots indicate individual donors. Median of the groups ± SD is given, *$p < 0.05$, Mann-Whitney test.
