# Supplementary material for: Immunotherapy-induced cytotoxic T follicular helper cells reduce numbers of retrovirus-infected reservoir cells in B cell follicles
Source: PLoS Pathog. 2023 Oct 26;19(10):e1011725. doi: 10.1371/journal.ppat.1011725 (PMC10602292; doi:10.1371/journal.ppat.1011725)
Supplement: S2 Fig — Representative dot plots of mWasabi-labeled FV-infected B cells (A) and Tfh cells (B) from lymph nodes were stained for MHC II expression on their surface. (PDF) [file ppat.1011725.s002.pdf]

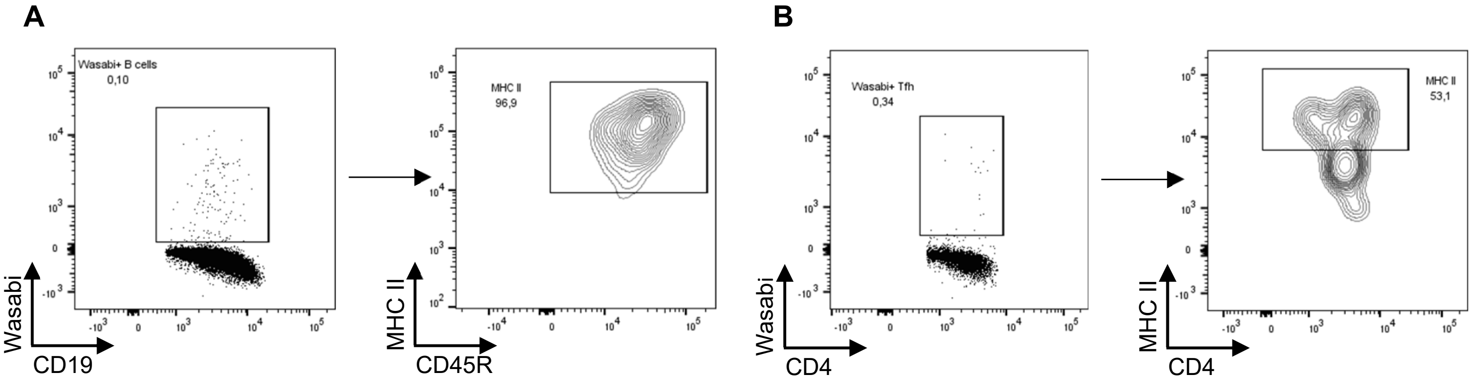

S2 Fig. FV-infected cells express MHC class II and can be targets of CD4-mediated killing.

Representative dot plots of mWasabi-labeled FV-infected B cells (A) and Tfh cells (B) from lymph nodes were stained for MHC II expression on their surface.
