# Supplementary material for: Immunotherapy-induced cytotoxic T follicular helper cells reduce numbers of retrovirus-infected reservoir cells in B cell follicles
Source: PLoS Pathog. 2023 Oct 26;19(10):e1011725. doi: 10.1371/journal.ppat.1011725 (PMC10602292; doi:10.1371/journal.ppat.1011725)
Supplement: S3 Fig — BALB/c mice were infected with FV, left untreated (gr1), ART-treated from 3–14 dpi (gr2), or ART-treated from 0–28 dpi (gr3) and sacrificed on 14 dpi (gr1, 2) or 28 dpi (gr3) (A). Single-cell suspensions from lymph nodes (LN), bone marrows (BM), and spleens were isolated and used for assessment of viral loads with an infectious center assay (B). Dots indicate values of individual mice. Median of the groups ± SD is given; dotted line DL–detection limit, **p < 0.01, ***p < 0.001, one-way ANOVA. (PDF) [file ppat.1011725.s003.pdf]

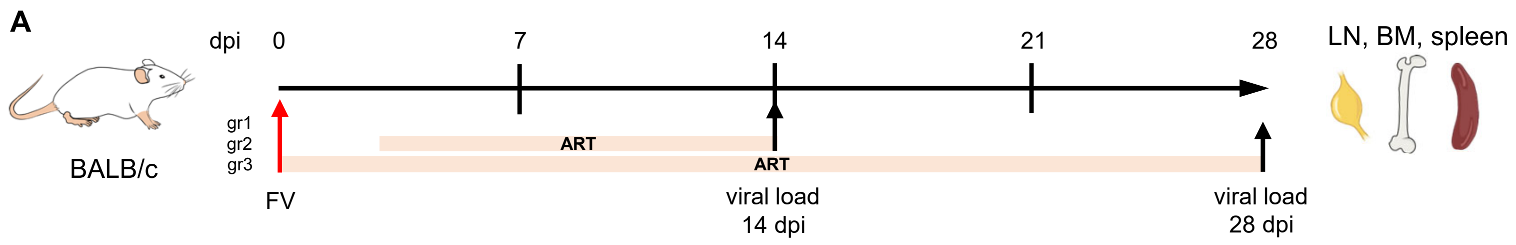

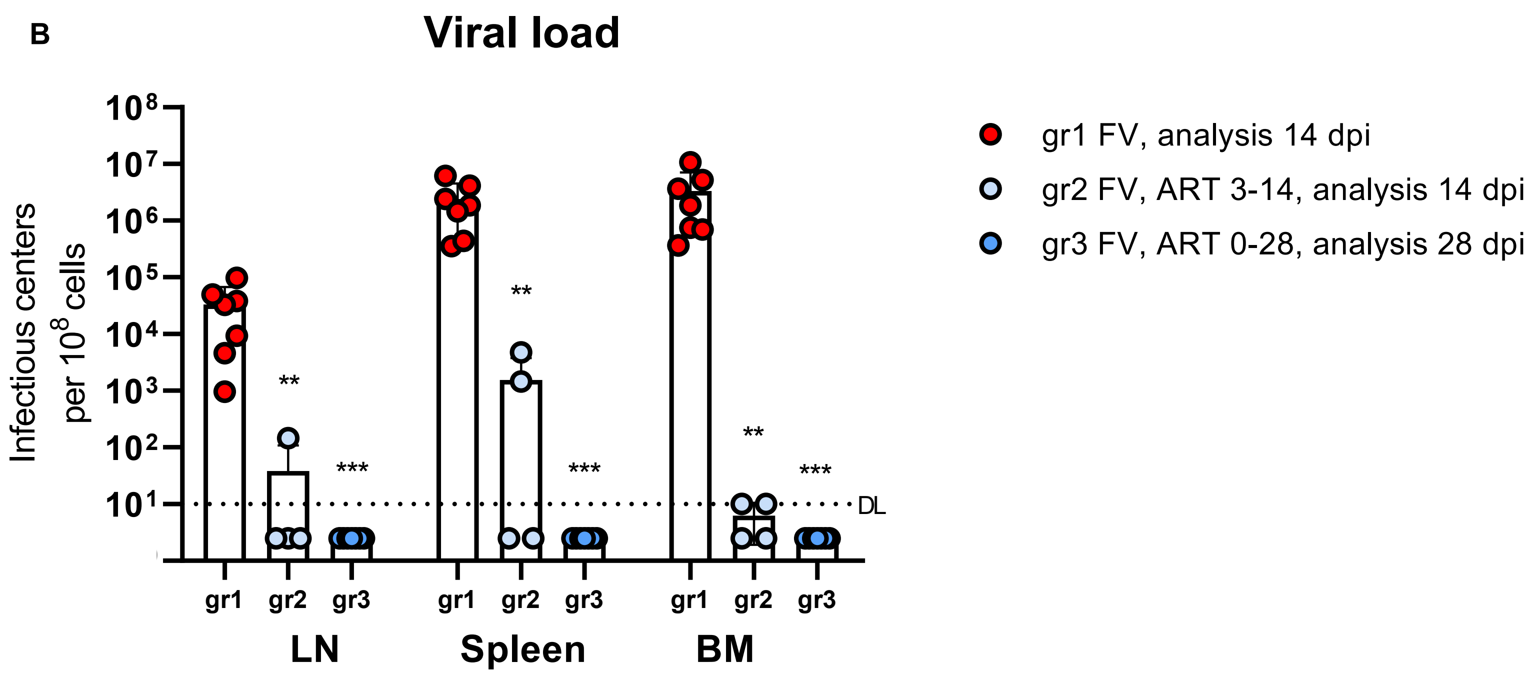

S3 Fig. ART suppresses FV infection in mice.

BALB/c mice were infected with FV, left untreated (gr1), ART-treated from 3-14 dpi (gr2), or ART-treated from 0-28 dpi (gr3) and sacrificed on 14 dpi (gr1, 2) or 28 dpi (gr3) (A). Single-cell suspensions from lymph nodes (LN), bone marrows (BM), and spleens were isolated and used for assessment of viral loads with an infectious center assay (B). Dots indicate values of individual mice. Median of the groups ± SD is given; dotted line DL – detection limit, $^{**}p < 0.01$, $^{***}p < 0.001$, one-way ANOVA.
