# Supplementary material for: Immunotherapy-induced cytotoxic T follicular helper cells reduce numbers of retrovirus-infected reservoir cells in B cell follicles
Source: PLoS Pathog. 2023 Oct 26;19(10):e1011725. doi: 10.1371/journal.ppat.1011725 (PMC10602292; doi:10.1371/journal.ppat.1011725)
Supplement: S4 Fig — CB6F1 mice were infected with mWasabi-expressing FV, treated with αCD137 (B and C) and/or ART (A and C). Dot plots from representative mice indicate the expression of cytotoxic molecules (Eomes and GzmB) by CXCR5+ PD1+ CD4+ T cells. (PDF) [file ppat.1011725.s004.pdf]

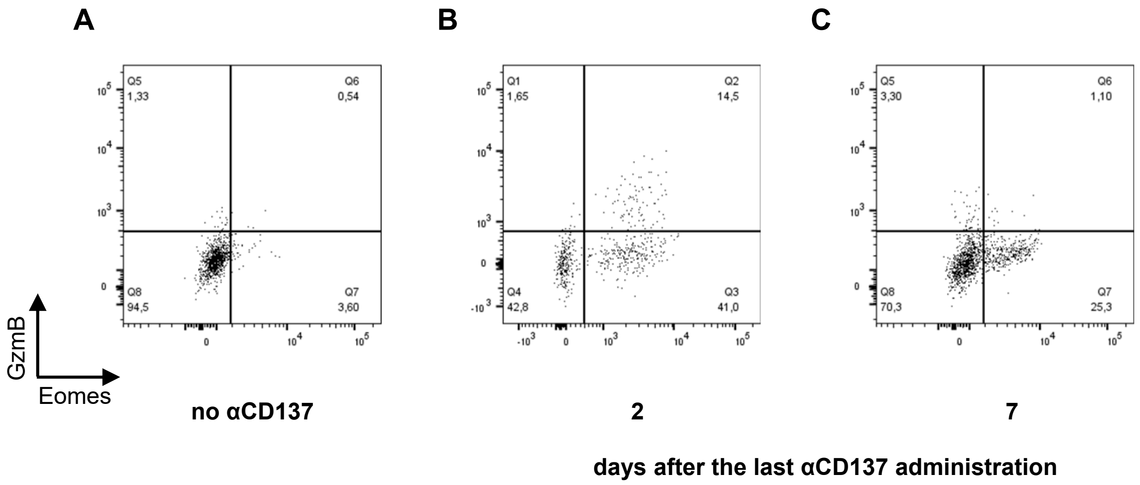

S4 Fig. Expression of cytotoxic markers of $CD4^+$ Tfh cells declines over time after the αCD137 injection.

CB6F1 mice were infected with mWasabi-expressing FV, treated with αCD137 (B and C) and/or ART (A and C). Dot plots from representative mice indicate the expression of cytotoxic molecules (Eomes and GzmB) by $CXCR5^+$ $PD1^+$ $CD4^+$ T cells.
